# Merck Open Global Health Library in vitro screening against *Schistosoma mansoni* identified two new substances with antischistosomal activities for further development

**DOI:** 10.1186/s13071-024-06648-0

**Published:** 2025-02-04

**Authors:** Monique Evelyn Ueberall, Martina Berchthold, Cécile Häberli, Sven Lindemann, Thomas Spangenberg, Jennifer Keiser, Christoph G. Grevelding

**Affiliations:** 1 Institute of Parasitology, BFS, Justus Liebig University Giessen, Giessen, Germany; 2 Department of Medical Parasitology and Infection Biology, Swiss Tropical and Public Health Institute, Allschwil, Switzerland; 3 University of Basel, Basel, Switzerland; 4 Merck Healthcare KgaA, Darmstadt, Germany; 5 Global Health R&D of the healthcare business of Merck KGaA, Darmstadt, Germany, Ares Trading S.A., (an affiliate of Merck KGaA, Darmstadt, Germany, Route de Crassier 1, 1262 Eysins, Switzerland

**Keywords:** *Schistosoma mansoni*, Schistosomiasis, Neglected tropical disease, Open Global Health Library, In vitro culture

## Abstract

**Background:**

Schistosomiasis, which is caused by the parasite *Schistosoma mansoni* as well as other species of the trematode genus *Schistosoma*, leads to chronic inflammation and finally to liver fibrosis. If untreated, the disease can cause life-threatening complications. The current treatment of schistosomiasis relies on a single drug, praziquantel (PZQ). However, there is increasing concern about emerging resistance to PZQ due to its frequent use.

**Methods:**

To identify potential alternative drugs for repurposing, the Open Global Health Library (OGHL) was screened in vitro, using two different screening workflows at two institutions, against adult *S. mansoni* couples and newly transformed schistosomula. This was followed by confirmation of the effects of the lead structures against adult worms.

**Results:**

In vitro screening at one of the institutions identified two fast-acting substances affecting worm physiology (OGHL00022, OGHL00121). The effects of the two lead structures were investigated in more detail by confocal laser scanning microscopy and 5-ethynyl 2´-deoxyuridine (EdU) assays to assess morphological effects and stem cell effects. Both substances showed negative effects on stem cell proliferation in *S. mansoni* but no further morphological changes. The EC_50_values of both compounds were determined, with values for compound OGHL00022 of 5.955 µM for pairing stability, 10.88 µM for attachment, and 18.77 µM for motility, while the values for compound OGHL00121 were 7.088 µM for pairing stability, 8.065 µM for attachment, and 6.297 µM for motility 24 h after treatment. Furthermore, *S. mansoni* couples were treated in vitro with these two lead structures simultaneously to check for additive effects, which were found with respect to reduced motility. The second in vitro screening, primarily against newly transformed schistosomula and secondarily against adult worms, identified four lead structures in total (OGHL00006, OGHL00022, OGHL00169, OGHL00217). In addition, one of the tested analogues of the hits OGHL00006, OGHL00169, and OGHL00217 showed effects on both stages.

**Conclusions:**

In two independent in vitro screening approaches against two stages of *S. mansoni* one common interesting structure with rapid effects was identified, OGHL00022, which provides opportunities for further development.

**Graphical Abstract:**

**Figure:**
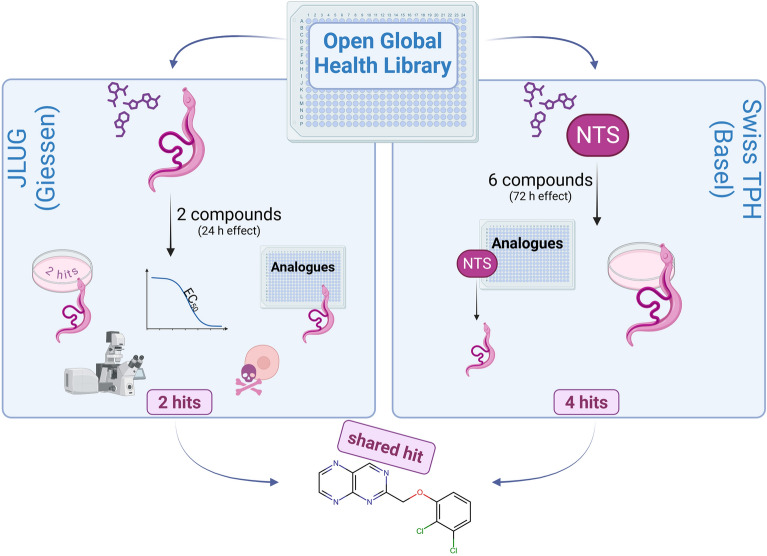

**Supplementary Information:**

The online version contains supplementary material available at 10.1186/s13071-024-06648-0.

## Background

Schistosomiasis is an infectious disease that affects humans and animals and is caused by parasites of the trematode genus *Schistosoma*. The World Health Organization has listed schistosomiasis as a neglected tropical disease (NTD), and estimates indicate that at least 251.4 million people required preventive treatment against this disease in 2021 [1, 2]. Climate change modelling and existing reports about the occurrence of schistosomiasis in southern Europe indicate that schistosomiasis may spread from tropical and subtropical areas to currently moderate climate zones, resulting in even more people facing the disease [3, 4]. There are several schistosome species that can infect humans, one of which is *Schistosoma mansoni* [2]. As with some other schistosome species, *S. mansoni* has zoonotic potential [5].

The complex life cycle of *S. mansoni* includes an intermediate freshwater snail host of the genus *Biomphalaria*, and humans or animals as final hosts [5, 6]. Miracidia hatch from the parasite’s eggs on contact with water and infect *Biomphalaria*, in which they develop via different sporocyst stages into cercariae. At the infectious stage, cercariae leave the snails and infect humans in contact with contaminated water. After penetrating the skin, cercariae develop into schistosomula, which migrate via the bloodstream to the liver, where they mature to the adult stage. Schistosomes are the only trematodes that have evolved different sexes, and pairing is prerequisite for female gonad differentiation and egg production [7]. After pairing, *S. mansoni* couples finally settle in the mesenteric veins of the gut and release eggs that pass through the gastrodermis, enter the gut lumen, and reach the environment in the faeces to continue the life cycle. Some of the eggs, however, remain inside the vascular system of the final host and are transported via the bloodstream to different organs, mainly the spleen and liver, where they induce inflammatory processes and fibrosis, which determine the pathology of schistosomiasis [2, 8, 9].

Preventive treatment plays a major role in fighting the disease. Besides intermediate host control and education programmes for affected human populations [9, 10], schistosomiasis control mainly relies on mass drug administration programmes using praziquantel (PZQ) in endemic areas for decades. Since 2007, millions of PZQ doses have been provided and administered to school-aged children and adults in sub-Saharan Africa [11, 12]. As model-based estimates indicate a decline in prevalence in MDA areas, PZQ seems to be a good weapon with which to control schistosomiasis [12]. A vaccine against schistosomiasis is still not available, and PZQ is the only drug used widely to fight the parasite, despite the fact that it cannot prevent reinfection. Furthermore, large-scale and frequent administration of PZQ increases the risk of reduced drug sensitivity, or eventually resistance of the parasite to it [13–15]. Therefore, it is necessary to find alternative treatment options [16, 17].

To address this issue, we undertook, and present the results of, two in vitro screening approaches using the Open Global Health Library (OGHL) from Merck against *S.* *mansoni* to identify potential lead structures for further optimization. The library is comprised of 250 diverse compounds from research and development, which consist of small molecules across a panel of human targets. The 250 compounds have 32 known distinct targets (eight compounds have unclear targets). More than half of the compounds are distributed across four known targets, which are type 5 phosphodiesterase (PDE5A), the primary target of sildenafil [18], a chemokine receptor (CCR2) involved in disease processes in various neurological disorders or autoimmune diseases [19], a Na^+^/H^+^ exchanger in mitochondrial membranes (NHE1) associated with cardiac dysfunction [20], and sphingosine 1 phosphate receptor-1, which is mainly involved in autoimmune diseases [21].

**Fig. 1 A, B:**
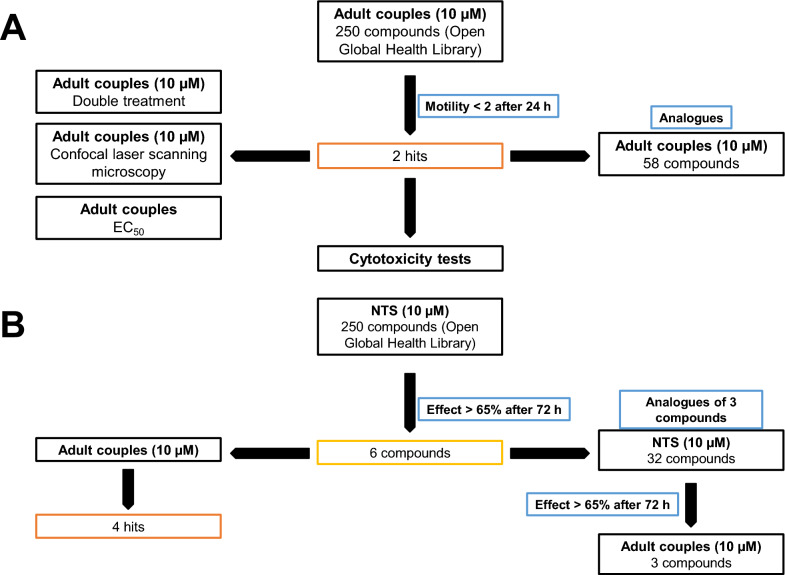
Open Global Health Library screening cascades against adult *Schistosoma mansoni*. **A** For the first screening cascade performed by the Giessen team, 250 compounds were initially screened against adult couples (10 µM). Two hits were identified using the criteria of a motility score below 2 after 24 h treatment (see below). Morphological effects of these two substances on adult *S. mansoni* were investigated by confocal laser scanning microscopy (CLSM) and determination of EC_50_ values. Additionally, couples were treated with both compounds at the same time to investigate additive effects. Finally, both substances were tested in cytotoxicity assays, and another smaller library of their analogues was screened against adult *S. mansoni* couples, again at a concentration of 10 µM for each compound. **B** For the second screening cascade, independently performed by the Basel team, the 250 compounds were screened against newly transformed schistosomula (NTS) at a concentration of 10 µM for 72 h, which identified six compounds (effect > 65%) for the next screening step. These six compounds were then tested against adult *S. mansoni* couples for 72 h (10 µM) and revealed four hits, which were also active against adults. In total, 32 analogues obtained from three of the hits (OGHL00006, OGHL00169, OGHL00217) followed the same screening cascade

The library was tested using two different test cascades (Fig. 1) in two different labs located at the Swiss Tropical and Public Health Institute (Swiss TPH) in Basel and at the Justus Liebig University Giessen (JLUG). Given the almost immediate effect of PZQ on adult schistosomes [22], the Giessen team aimed to find compounds with rapid activity against adult *S.* *mansoni* in an established in vitro culture system [23]. Of the compounds tested, two showed rapid activity (within 24 h) against the adult stage of the parasite. In a second approach, the Basel team used newly transformed schistosomula (NTS) to pre-screen the compounds, of which the best were additionally tested against adult worms. This approach identified four compounds with effects on both stages.

## Methods

### Maintenance of the *S. mansoni* life cycle

To maintain the life cycle of the *S. mansoni* at the JLUG and Swiss TPH, *Biomphalaria glabrata* snails were used as the intermediate host and the golden Syrian hamster *Mesocricetus auratus* (JLUG; strain RjHan:AURA was used at the Swiss TPH) served as the final host [24]. Snails were maintained in aquaria at 26 °C and a day/night cycle of 16 h light and 8 h dark. For infection, individual snails were infected with several miracidia of an isolate of the Liberian strain of *S. mansoni* [25]. After 28 days, cercarial shedding was induced by exposing the snails to light. The cercariae were then used to infect hamsters (8 weeks old) as the final host. Adult worms were harvested at 46 days post-infection by hepatoportal perfusion. Miracidia were recovered from eggs extracted from the livers of the infected hamsters to infect new snails [25].

#### Ethics statements

Experiments with hamsters to obtain *S. mansoni* worms and eggs were performed in accordance with the European Convention for the Protection of Vertebrate Animals Used for Experimental and Other Scientific Purposes (ETS no. 123). Animal experiments performed by the Giessen team were approved by the Regional Council (Regierungspräsidium) Giessen (V54-19 c 20/15 h 02 GI 18/10 no. A 26/2018). In vitro studies carried out by the Basel team were performed in line with the Swiss national and cantonal regulations in animal welfare at the Swiss Tropical and Public Health Institute (Basel-Landschaft, Switzerland) under permission number 520.

### Preparation of parasites and in vitro cultures

For in vitro culture in Giessen, M199 medium (Gibco) containing 10% newborn calf serum, 1% HEPES (1 M) and 1% ABAM solution (10,000 units penicillin, 10 mg streptomycin and 25 µg amphotericin B per millilitre) were used [24, 26]. After perfusion, the worms were transferred to petri dishes with 5 mL medium using featherweight tweezers. The worms were cultured at 37 °C and 5% CO_2_. After 24 h, stably paired couples were selected for inhibitor assays.

At the Basel lab, infected *B. glabrata* snails were placed for 4 h under a light source to initiate the shedding of cercariae, which were collected, mechanically transformed into NTS, and kept in M199 medium supplemented with 5% fetal calf serum (FCS), 1% Mäsermix, and 1% penicillin/streptomycin (pen./strep.) 10,000 U/ml, at 37 °C and 5% CO_2_ [27]. To collect adult *S.* *mansoni*, mesenteric veins of infected hamsters were dissected on day 49 post-infection. The worms were placed in supplemented RPMI 1640 medium (containing 5% FCS, 1% pen., and 100 μg/ml strep.) and incubated at 37 °C with 5% CO_2_ until further use.

### Inhibitor assays

The 250 compounds of the OGHL (Merck Healthcare KgaA, Darmstadt, Germany) were provided to both labs (Basel and Giessen), dissolved in 30 µL DMSO at a concentration of 10 mM and stored at − 20 °C. For secondary screening, the Giessen team received 58 additional analogues of the compounds OGHL00022 and OGHL00121, and for further tests, an additional 3 mg of the compounds OGHL00022 and OGHL00121 were provided and dissolved in pure DMSO at a concentration of 10 mM, and also stored at − 20 °C. The Basel team obtained 32 analogues of the compounds OGHL00006, OGHL00169, and OGHL00217 for secondary screening.

Figure 1 provides the respective screening cascades and the inhibitor assays performed in each lab and summarizes initial screenings at different concentrations of each compound, different time points, different life stages, further investigations of hit compounds, and first cytotoxicity tests.

#### Screening cascade and double treatment in Giessen

The initial screening by the Giessen team covered the 250 compounds of the OGHL, and in a subsequent, second screening approach, an additional 58 analogues of the compounds OGHL22 and OGHL121, which were tested on *S. mansoni* couples in vitro. For each compound, three replicates of five couples randomised from different hamsters were used. The couples were transferred to 6-well plates (or 12-well plates) containing 2 mL pre-heated medium. For each compound, the stock solution was pre-diluted in pre-heated medium, and then added to each replicate, resulting in a final volume of 2.5 mL and a final concentration of 10 µM. A negative control was included for all assays. For this, three replicates were prepared with DMSO (0.1%) as volume substitution of the stock solution. The worms were cultured at 37 °C and 5% CO_2_. As physiological parameters, worm motility, pairing stability, and attachment were assessed after 4 h and 24 h. After 24 h, egg production was also estimated (see below).

For the compounds OGHL00022 and OGHL00121, a double treatment was performed in a similar manner, but with three exceptions. Instead of three replicates, each with five worm pairs randomised from different hamsters, the double treatment was performed with worms from three hamsters as a biological triplicate, as was done for determining the EC_50_ values. Furthermore, double the amount of DMSO corresponding to the DMSO concentration of two added stock solutions was used as a negative control, and egg numbers were counted.

#### Screening cascade in Basel

The Basel team performed the screening as follows: the stock solutions of each compound from the library (10 mM) were diluted with M199 medium (supplemented with 1% pen./strep., 1% Mäsermix, 5% FCS) and NTS solution (0.7 NTS/µL) in a transparent flat-bottom 96-well plate (Sarstedt, Switzerland), resulting in a final volume of 250 µL, 30–40 NTS, and a drug concentration of 10 µM. For adults, the drugs were diluted to 10 µM in 2 mL with RPMI 1640 medium (supplemented with 1% pen./strep. and 5% FCS) in a 24-well plate (Sarstedt, Switzerland), and three schistosome couples were added. Compounds revealing activity (effect > 65%) were tested at 1 µM and 0.1 µM, and also evaluated after 24 h. For both life stages, DMSO served as a negative control (< 1% of the final volume). The plates were incubated at 37 °C and 5% CO_2_. All compounds were tested in triplicate for NTS or duplicate for adults.

#### Determination of EC_50_ values

For two compounds, OGHL22 and OGHL121, the Giessen team determined EC_50_ values for pairing stability, attachment and motility of schistosome couples after 4 h and after 24 h. For this purpose, three replicates of five couples per concentration were prepared in 2 mL pre-heated medium. For each biological replicate, five couples were obtained from one hamster. For each concentration, a predilution was prepared with medium and the appropriate volume of stock solution. The predilution was then added to the culture, resulting in final concentrations of 2.5 µM, 5 µM, 10 µM, 20 µM, and 50 µM, and a final volume of 2.5 mL. As a negative control, DMSO was added according to the highest concentration. The worms were cultured at 37 °C and 5% CO_2_ and scored after 4 h and after 24 h.

### Assessment of viability and egg production

For the first screening approach, the Giessen team evaluated pairing stability by counting couples at the time of scoring. Attachment was determined by counting the number of worms (couples or single worms) that were attached to any surface. Motility was scored on a scale of 0–4. The average activity of the worms in the negative control was used as a reference value for normal activity: 4 indicated increased activity, 3 normal activity, 2 reduced activity, 1 little activity with occasional head and tail movements and/or gut movement, 0 no activity for at least 20 s. The number of eggs was estimated after 24 h. Compared to the control, categories for egg production were defined as fewer eggs (score 0), similar numbers of egg (score 1) and more eggs (score 2). The corresponding percentages of the scores were determined based on three replicates. Score 0 corresponded to less than 50% eggs, score 1 to 50–200%, and score 2 to over 200% of the egg count compared to the negative control. The means of the scores were later normalized to the range 0–2 (0–200%).

The Basel team checked viability after 72 h through visual inspection as described by Lombardo et al. [27] using a viability scale of 0.25 scoring steps.

### Morphological analyses and 5-ethynyl 2´-deoxyuridine assays

For morphological analyses of schistosome couples treated with compounds OGHL00022 and OGHL00121 (10 µM each), the Giessen team performed confocal laser scanning microscopy (CLSM). Instead of three replicates with five pairs of randomised hamsters each, the treatment was carried out with five worms from three hamsters as biological triplicates. After 24 h following one-time treatment with each compound, worm couples were separated with tricaine, fixed in AFA (95% EtOH, 3% formaldehyde, 2% glacial acetic acid), and stored at 4 °C. The worms were then stained in carmine red solution for 45 min at room temperature and destained in acidic EtOH for 5 min. Worms were then dehydrated successively in 90% EtOH for 5 min and 100% EtOH for 5 min and mounted on slides with Euparal [28]. An inverted CLSM (Leica TSC SP5; Leica, Germany) was used to image stained worms. Carmine red was excited by using an argon laser at 488 nm.

To investigate potential effects on stem cell proliferation, the Giessen team performed 5-ethynyl-2′-deoxyuridine (EdU; a thymidine analogue) assays as described before [29]. To this end, schistosome couples were first treated with the compounds OGHL00022 and OGHL00121 once (10 µM). After 24 h, the worms were incubated for a further 24 h with 10 μM EdU, using the Click-it Plus Edu Alexa Fluor 488 Imaging Kit (Thermo Fisher Scientific). Subsequently, the couples were separated using tricaine, fixed, and stained as described before [30]. After counterstaining with Hoechst 33342 (8 µM), the worms were mounted on slides using ROTImount FluoCare (Carl Roth). For the EdU incorporation assay, Alexafluor488 was excited at 488 nm, and Hoechst 33342 was excited at 405 nm.

### Tests for characteristic parameters

To determine the kinetic solubility, the compounds dissolved in DMSO were measured in PBS at pH 7.4 after 2 h using HPLC analysis (filtrate). To predict the oral in vivo absorption of the compounds, Caco-2 permeability assays were conducted. For this purpose, the Caco-2 cell line (wild type, passage number 40–60, cultivated for 21 days in 24-well plates) was used. The incubation time was 2 h at 37 °C, with atenolol (low passive permeability) and metoprolol (high passive permeability) serving as controls. The compounds were tested at a concentration of 10 µM in triplicate. Furthermore, metabolic stability was assessed using intrinsic clearance assays. Finally, the IC_50_ values of the target enzymes were determined.

### Data analysis

Means and SDs were calculated using Microsoft Excel. Heat maps and graphs were generated using GraphPad Prism (Dotmatics). This software was also used for further statistical analyses, e.g. paired* t*-tests (95% confidence level with two-tailed* p*-value), and to determine and plot EC_50_ values using the non-linear regression model with the equation: [inhibitor] vs. response–variable slope (four parameters). The variables were automatically defined and only fixed when necessary to 0% (bottom) and 100% (top) for pairing stability and attachment. CLSM images were edited using ImageJ (Fiji package) [31]. Chemical structures were visualized using Marvin for JavaScript (Chemaxon).

## Results

### First in vitro studies of OGHL compounds against adult *S. mansoni* by the Giessen team identified two fast-acting substances affecting worm physiology

**Fig. 2:**
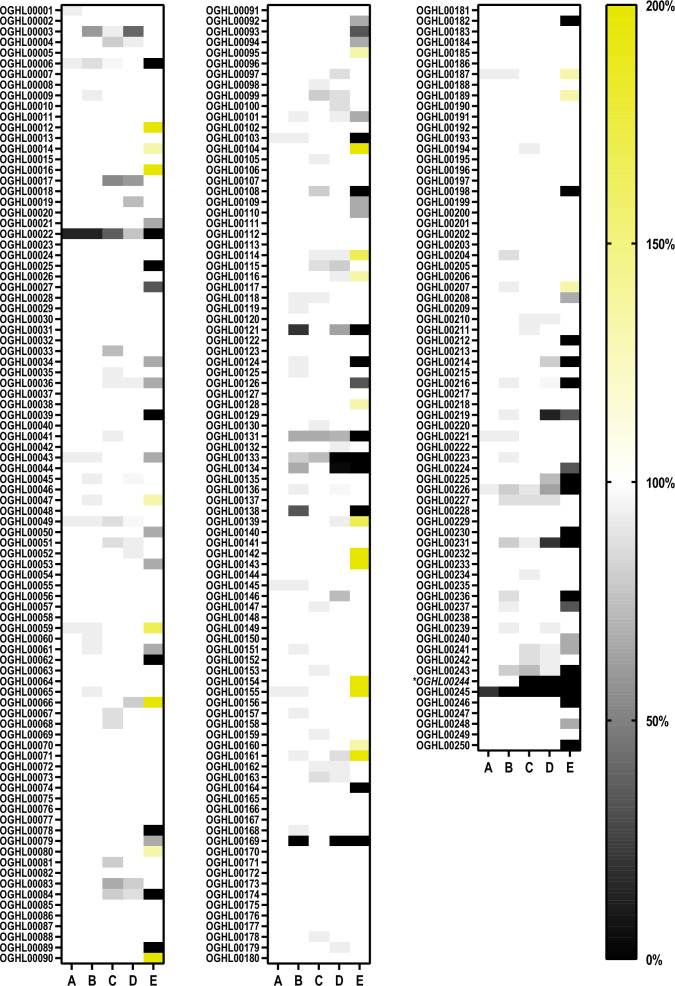
In vitro screening results of the OGHL on physiological parameters of adult *Schistosoma mansoni.* Adult *S. mansoni* were treated with 10 µM of each compound by the Giessen team. The results are summarised in a heat map, in which the scales for paired worms and attached worms are given from 0% (black) and 100% (white). The scale is extended to 200% (yellow) for the normalised egg score compared to the negative control. *A*, *B* Pairing stability (%) after 4 h and 24 h of treatment, respectively;* C*, *D* attachment capacity (%) after 4 h and 24 h of treatment, respectively;* E* egg production score (%) in comparison to the negative control (DMSO only) after 24 h of treatment. OGHL00244 [(*R*)-PZQ] is given in italics and marked with an asterisk

**Fig. 3:**
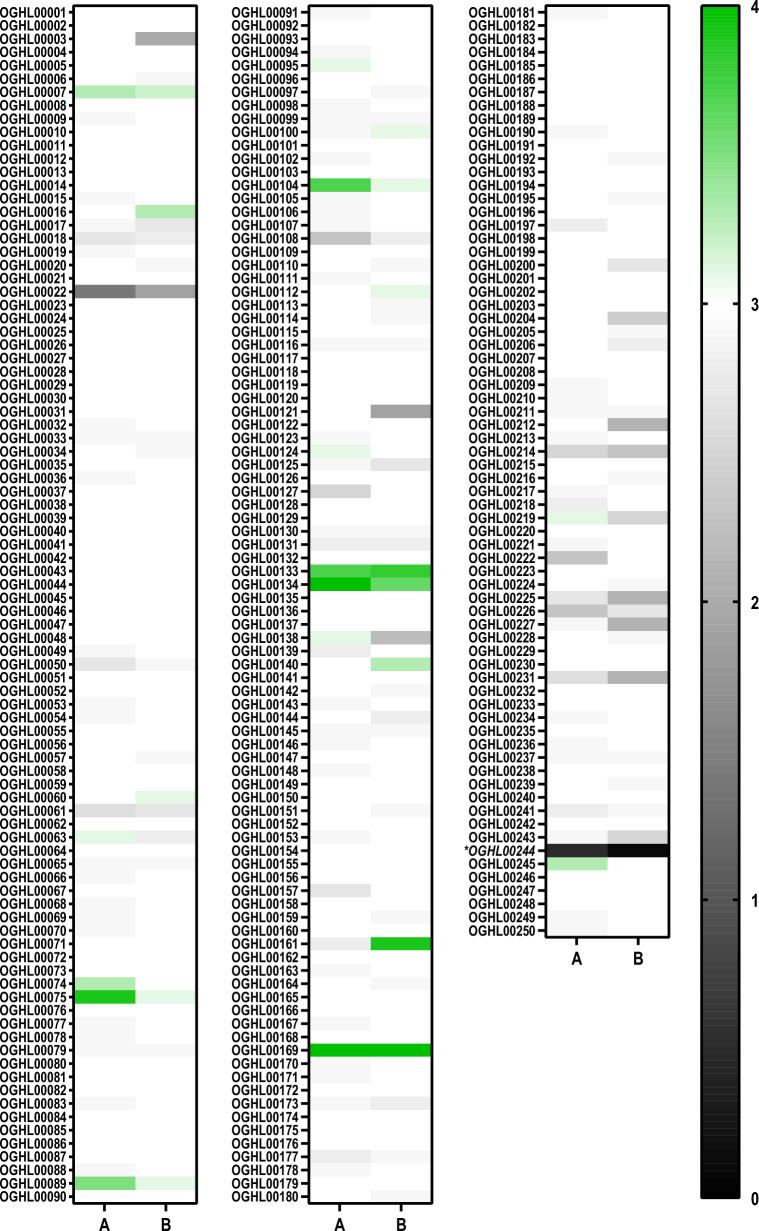
In vitro screening results of the OGHL on the motility of adult *Schistosoma mansoni.* Adult *S. mansoni* were treated with 10 µM of each compound by the Giessen team. The results are summarised in a heat map, in which the motility score 0 (no motility) is given in black; it gradually transitions through shades of grey to white for weak (score 1–2) to normal motility (score 3), and increased motility (score 4), which is given in green.* A*,* B* Motility scores after 4 h and 24 h of treatment, respectively. OGHL00244 [(*R*)-PZQ] is given in italics and marked with an asterisk

For the initial screening by the Giessen team, the 250 OGHL compounds were tested for their effects on *S. mansoni* couples in vitro using a concentration of 10 µM per compound. As physiological effects, pairing stability of couples, attachment capacity, and egg production (Fig. 2) were investigated, as well as motility (Fig. 3). Substance-treated and DMSO-treated (control) worms were monitored after 4 h and 24 h with a focus on rapid effects. The concentration of 10 µM was chosen because it is in the range of the effective concentration of PZQ in vitro [32, 33].

Compound OGHL00244 ((*R*)-PZQ), which is included in the OGHL and acted as a blinded positive control, already showed the strongest negative effect on motility and strong effects on attachment capacity after 4 h. Although OGHL00244 showed no effect on pairing stability at these two timepoints, the number of eggs was drastically reduced (after 24 h). Following the first in vitro screen, these results validated the reliability of the in vitro screening cascade.

Of the 250 compounds tested by the Giessen team, 13 already showed effects on pairing stability of couples after 4 h (pairing stability < 100%). Two of the compounds, OGHL00022 and OGHL00245, showed strong effects on pairing stability: > 50% of the couples had already separated after 4 h. After 24 h, 46 compounds caused effects on pairing stability, five of which resulted in the separation of > 50% of the couples. OGHL00169 and OGHL00245 caused the separation of all couples.

OGHL compounds also affected the attachment capacity of worms. After 4 h, 44 compounds showed detachment effects (attachment < 100%), with three compounds causing detachment of > 50% of the worms. After 24 h, the number of compounds causing detachment increased to 47, of which eight caused detachment of > 50% of the worms. Although many compounds showed effects on both pairing stability and attachment, not all of them affected these two parameters simultaneously.

Of note, 79 compounds of the library (31.6%) affected egg production. While 23 compounds caused increased egg production, 56 caused its reduction after 24 h (Additional file 1).

In total, 95 compounds influenced worm motility (motility score ≠ 3) after 4 h. Of these, 14 caused an increase in worm activity (motility score > 3). The remaining 81 reduced worm movement (motility score < 3). Of these, two compounds exhibited strong reduction effects (motility score < 2) after 4 h, OGHL00022 (motility score of 1.4) and OGHL00244 (PZQ; motility score of 0.5).

After 24 h, 74 compounds showed effects on worm motility (motility score ≠ 3). Of these, 13 increased worm activity (motility score > 3), whereas 61 reduced worm activity (motility score < 3). In addition to the two fast-acting (4 h) compounds, another compound, OGHL00121, caused a strong effect (motility score < 2) on worms after 24 h. Because of their strong effects on worm motility after 24 h, compounds OGHL00022 and OGHL00121 [motility score of 1.9 for both compounds (Table 1)] were selected for further investigation.

### First in vitro studies of OGHL compounds against NTS by the Basel team identified six compounds for further tests on adult *S. mansoni* in the second screening approach

**Fig. 4:**
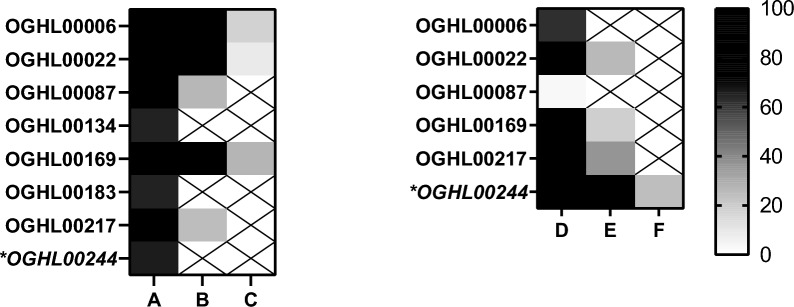
In vitro screening results of the OGHL compounds with effects on NTS and adult *Schistosoma mansoni.* NTS and adult *S. mansoni were* treated with concentrations between 0.1 µM and 10 µM by the Basel team. For the heat map, the scale is given for the compound effect from 0% (white) to 100% (black).** A**,** B**,** C** Effects on NTS after 72 h of treatment with 10 µM, 5 µM, and 1.25 µM of each compound, respectively.** D**,** E**,** F** Effects on couples after 72 h of treatment with 10 µM, 1 µM, and 0.1 µM of each compound, respectively. OGHL00244 [(*R*)-PZQ] is given in italics and marked with an asterisk

The Basel team tested the 250 compounds also at the concentration of 10 µM each against NTS using an incubation period of 72 h. As shown in Fig. 4, six compounds revealed an activity of greater than 65% (OGHL00006, OGHL00022, OGHL00087, OGHL00169, OGHL00217 and OGHL00244 [(*R*)-PZQ]). Of these, OGHL00006 and OGHL00087 caused the death of all worms. OGHL00183 and OGHL00134 showed an activity of 65% and were not considered further. Next, the six compounds were tested on adult worms. Except for OGHL00087, which revealed no activity, four compounds and [(*R*)-PZQ] showed activity on adult worms, ranging from 62 to 95% after incubation for 72 h (OGHL00006, OGHL00022, OGHL00169, OGHL00217, OGHL00244 [(*R*)-PZQ]). The structures of the hits are shown in Tables 1 and 2. Moderate activity of 46–55% was observed on incubating OGHL00006, OGHL00022, OGHL00169, OGHL00217 at 10 µM for 24 h. Hence, none of the four hits were studied further.

### EC_50_ values of OGHL00022 and OGHL00121 showed antischistosomal activities in low millimolar ranges

**Fig. 5 A–F:**
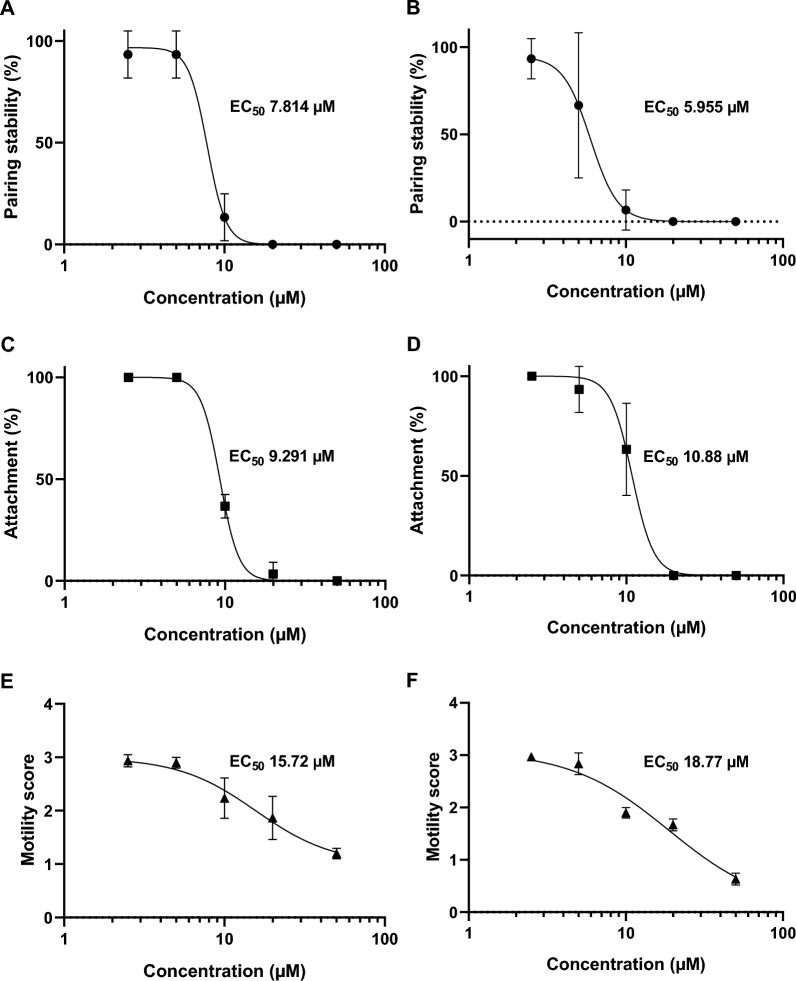
EC_50_ values of OGHL00022. EC_50_ values of OGHL00022 with corresponding graphs for pairing stability (●), attachment (■), and motility (▲) of adult *Schistosoma mansoni* shown over different concentrations (2.5–50 µM) by the Giessen team. **A**, **B** Pairing stability (%) after 4 h and 24 h of treatment, respectively. **C**, **D** Attachment (%) after 4 h and 24 h of treatment, respectively. **E**, **F** Motility score after 4 h and 24 h of treatment, respectively. Shown are the means and SDs (*n* = 3)

**Fig. 6A–F:**
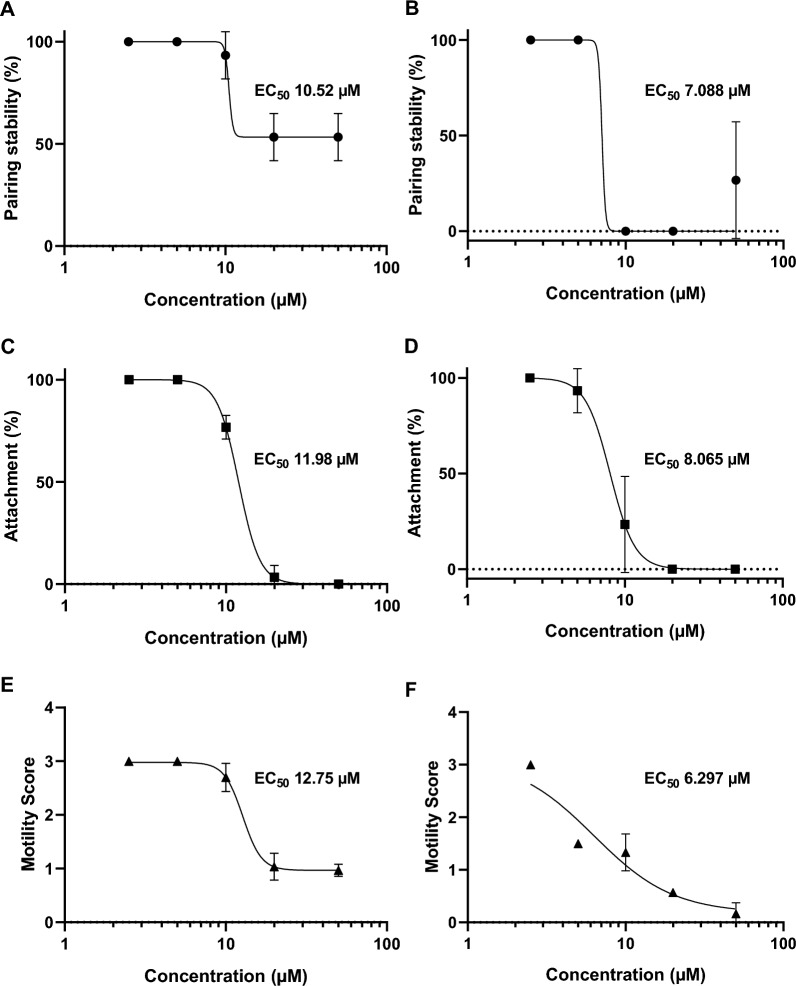
EC_50_ values of OGHL00121. EC_50_ values of OGHL00121 with corresponding graphs for pairing stability (●), attachment (■) and motility (▲) of adult *Schistosoma mansoni* shown over different concentrations (2.5–50 µM) by the Giessen team. **A**, **B** Pairing stability (%) after 4 h and 24 h of treatment, respectively. **C**, **D** Attachment (%) after 4 h and 24 h of treatment, respectively. **E**, **F** Motility score after 4 h and 24 h of treatment, respectively. Shown are the means and SDs (*n* = 3)

For the two selected OGHL compounds showing strongest motility effects in the Giessen lab, OGHL00022 and OGHL00121, EC_50_ values were determined for pairing stability, attachment capacity, and motility after 4 h and 24 h by the Giessen team (Figs. 5, 6; Table 1).

For pairing stability, the EC_50_ values of compound OGHL00022 were 7.814 µM at 4 h and 5.955 µM at 24 h after treatment. For attachment, the EC_50_ values were 9.291 µM at 4 h and 10.88 µM at 24 h after treatment. The value for motility increased from 15.72 µM at 4 h to 18.77 µM at 24 h. The EC_50_ value of compound OGHL00121 for pairing stability was 10.52 µM at 4 h and decreased to 7.088 µM at 24 h after treatment; for attachment, it was 11.98 µM at 4 h and 8.065 µM at 24 h. The value for motility also decreased, from 12.75 µM at 4 h to 6.297 µM at 24 h.

### OGHL00022 and OGHL00121 showed effects on cell proliferation in *S. mansoni*

**Fig. 7:**
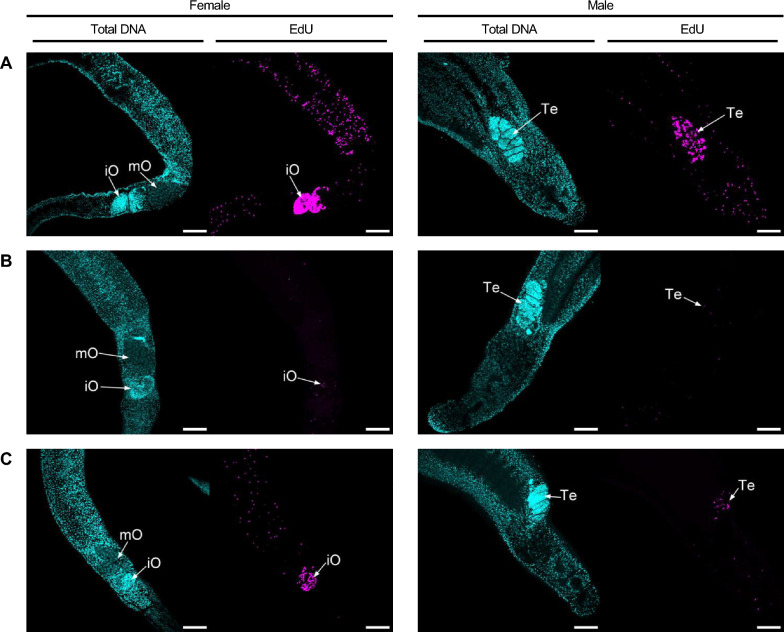
Influence of OGHL00022 and OGHL00121 on stem cell proliferation in *Schistosoma mansoni* couples. Representative images showing worms treated for 24 h with DMSO (control; **A**), 10 µM OGHL00022 (**B**), or 10 µM OGHL00121 (**C**), before the EdU assays were performed. Total DNA stained with Hoechst is shown in cyan, and EdU-positive cells are shown in magenta. Part of the ovary containing immature oocytes (*iO*); part of the ovary containing mature oocytes (*mO*).* Te*, Testes. Scale bars represent 100 µm

Against the background of the importance of stem cells for the biology of parasites such as *S. mansoni* [34], the Giessen team investigated stem cell proliferation using EdU assays in *S. mansoni* couples following treatment (24 h) with OGHL00022 and OGHL00121 (Fig. 7). EdU-positive cells are representative of somatic stem cells (neoblasts) and gonadal stem cells undergoing cell division [35]. Worms treated with OGHL00121 showed a complete reduction of EdU-positive cells in the testes, and a slight reduction in the ovaries, while worms treated with OGHL00022 showed almost no EdU signals in either males or females.

**Fig. 8 A, B:**
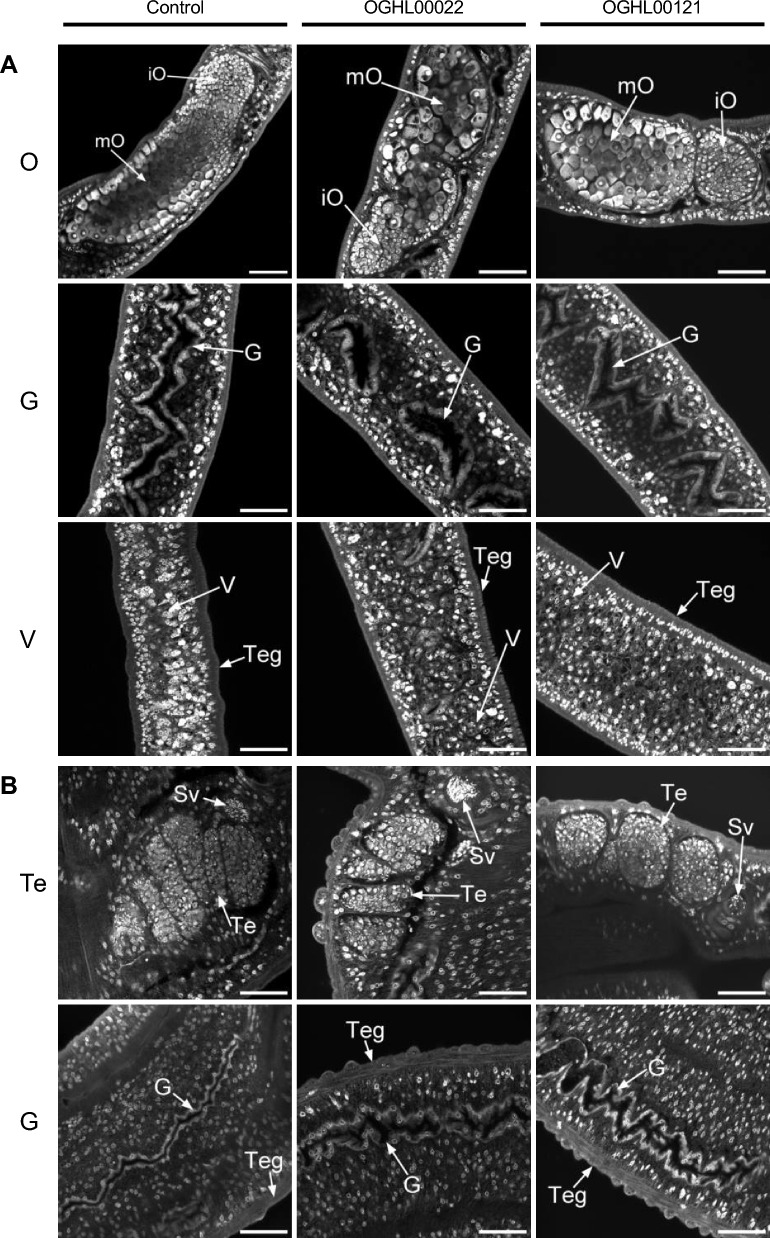
No clear morphological changes in *Schistosoma mansoni* couples after 24 h of treatment with OGHL00022 and OGHL00121. The worms were treated with 10 µM DMSO (control), 10 µM OGHL00022, or 10 µM OGHL00121. Shown are representative images of ovary (*O*), gut (*G*), and vitellarium (*V*) of females (**A**), and Te and gut (*G*) for males (**B**) after separation.* Teg* Tegument,* Sv* seminal vesicle; for other abbreviations, see Fig. 7. Scale bars represent 50 µm

To assess morphological effects of the compounds 24 h after treatment, the worms were studied by CLSM with a focus on the tegument, the gut, and the gonads (Fig. 8) by the Giessen team. At this time point, no clear morphological changes were observed after treatment with either compound.

### OGHL00022 and OGHL00121 double treatment caused an additive motility effect in adult *S. mansoni*

To test for additive effects of the two most potent compounds, the Giessen team treated *S.* *mansoni* couples simultaneously with OGHL00022 and OGHL00121.

**Fig. 9 A–D:**
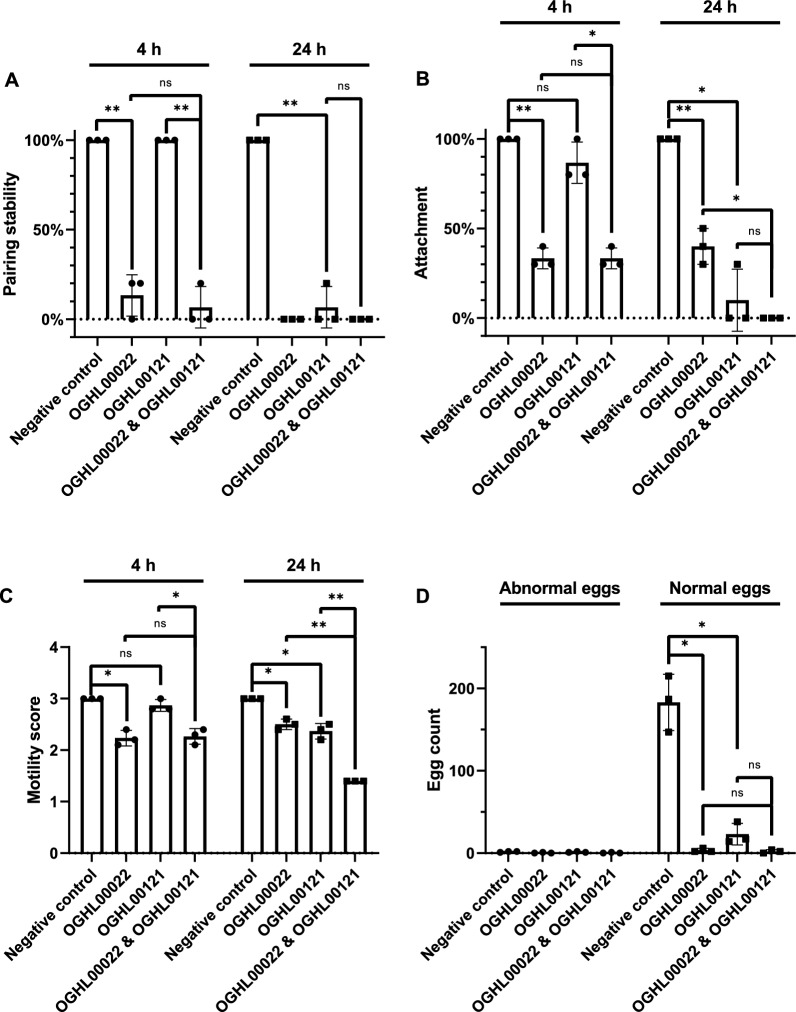
In vitro results of single and double treatments with OGHL00022 and/or OGHL00121. *Schistosoma mansoni* couples were treated with 10 µM of OGHL00022 and/or OGHL00121 by the Giessen team. Pairing stability, attachment capacity, and motility were determined after 4 h (●) and after 24 h (■), as well as egg numbers after 24 h of treatment. **A** Pairing stability (%), **B** attachment (%), **C** motility score, **D** numbers of normal and abnormal eggs. Shown are the values for each biological replicate, the means, and SDs (*n* = 3). Two-tailed *t*-tests were performed with values for motility obtained after 24 h (each single treatment vs double treatment); ns *p* > 0.05, * *p* ≤ 0.05, ** *p* ≤ 0.01

No additive effect were observed on pairing stability, as OGHL00022 alone caused complete separation after 24 h of treatment (Fig. 9). Similarly, double treatment failed to destabilize attachment capacity, as OGHL00121 alone caused detachment of all worms after 24 h. However, an additive effect was observed for motility after 24 h. Here, the motility score of worms was 1.4 ± 0.0, whereas single treatments with OGHL00022 and OGHL00121 led to motility scores of 2.5 ± 0.1 and 2.4 ± 0.2, respectively (each* n* = 3).

As only OGHL00022 almost completely inhibited egg production, it was not possible to determine an additive effect on this parameter upon double treatment.

### OGHL00022 and OGHL00121 analogues showed no strong effects against adult *S. mansoni *in vitro

**Fig. 10:**
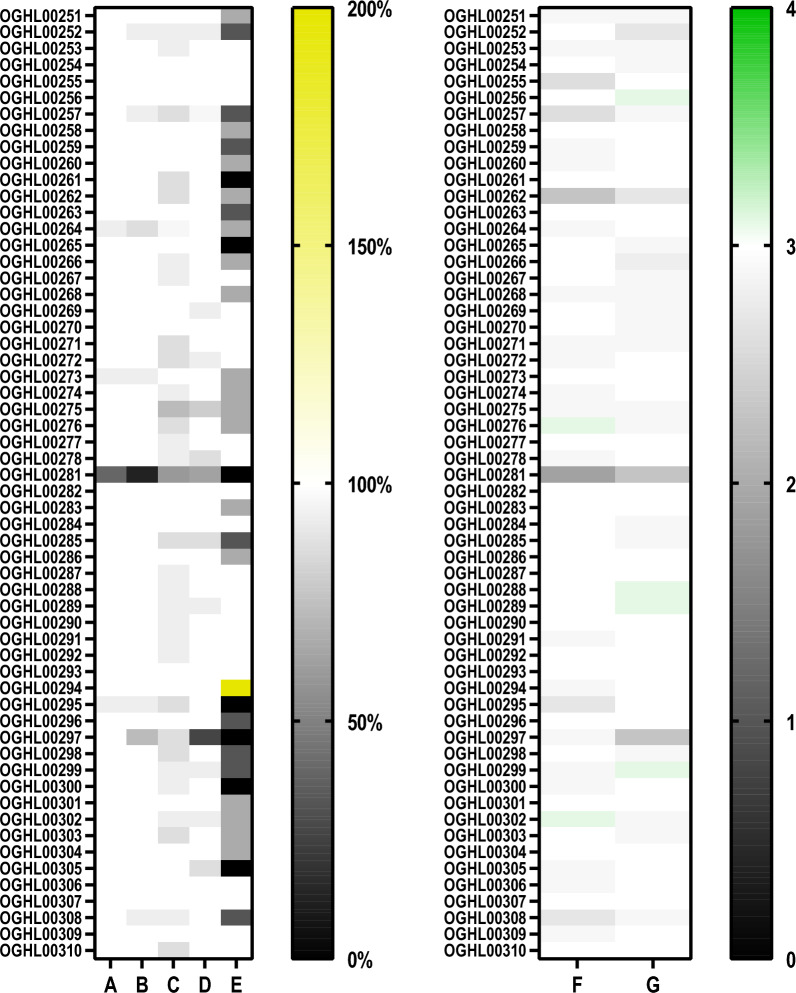
In vitro screening results of 58 analogues of OGHL22 (OGHL251-278) and OGHL121 (OGHL 281-310). Adult *Schistosoma mansoni* were treated with 10 µM of each analogue by the Giessen team. The results for pairing stability and attachment are summarised in the left heat map, in which the scales for paired worms and attached worms are given from 0% (black) and 100% (white). The scale is extended to 200% (yellow) for the normalised egg score compared to the negative control. In the right heat map, the motility score 0 (no motility) is given in black; it gradually transitions through shades of grey to white for weak (score 1–2) to normal motility (score 3), and an increased motility score, which is given in green (score 4).** A**,** B** Pairing stability (%) after 4 h and 24 h of treatment, respectively.** C**,** D** Attachment capacity (%) after 4 h and 24 h of treatment, respectively.** E** Egg production score (%) in comparison to the negative control (DMSO only) after 24 h of treatment.** F**,** G** Motility score after 4 h and 24 h of treatment, respectively

Because of the promising effects observed for OGHL00022 and OGHL00121, the Giessen team additionally tested 58 analogues of these two compounds (Fig. 10).

Four of the analogues showed effects on pairing stability after 4 h, and eight destabilized couples after 24 h. Of these, only OGHL00281 (analogue of OGHL00121) induced couple separation > 50% after 4 h and 24 h.

After 4 h, 32 analogues decreased worm attachment, none of which led to > 50% detachment. After 24 h, 13 analogues influenced attachment, one of which,OGHL00297 (analogue of OGHL00121), showed a detachment rate > 50%.

After 4 h, 27 analogues affected worm motility (motility score ≠ 3). Of these, two caused an increase in worm movement (motility score > 3). The remaining 25 analogues reduced worm movement (motility score < 3). Only one had a strong effect (motility score < 2) after 4 h, OGHL00281 (motility score of 1.9). After 24 h, however, the treated worms showed a motility score of 2.3 once again. In total, 28 analogues affected worm motility after 24 h (motility score ≠ 3), four of which showed an increase in activity (motility score > 3). No analogue had a strong effect on worm motility after 24 h (motility score < 2). Furthermore, 34 out of the 58 analogues (58.6%) caused a reduction of egg production; however, none caused its increase within 24 h.

### Characteristic parameters for OGHL00022 and OGHL00121

No in vivo data were collected for OGHL00022 and OGHL00121; however, their kinetic solubility was determined. OGHL00022 exhibited a kinetic solubility of 170 µM (PBS pH 7.4, 2 h), while OGHL00121 showed a solubility of 150 µM. The Caco-2 permeability for OGHL00022 was measured from apical to basolateral at 24.3 × 10^−6^ cm/s and from basolateral to apical at 20.9 × 10^−6^ cm/s, resulting in an efflux ratio of 0.86, indicating that the compound does not undergo active efflux. For OGHL00121, the permeability from apical to basolateral was 0.080 × 10^−6^ cm/s and from basolateral to apical was 0.22 × 10^−6^ cm/s, yielding an efflux ratio of 2.75, suggesting active efflux.

Additionally, the metabolic stability of OGHL00121 was assessed by determining the intrinsic clearance in mouse and human liver cells, with values of 42.0 µl/min per mg protein (mouse, 1 µM, 30 min incubation) and 20.0 µl/min per mg protein (human, 1 µM, 30 min incubation), respectively.

The human target for OGHL00022 is CX3CR1, with the compound exhibiting an IC_50_ value of 135 nM in cellular CX3CR1 assays. The primary target for OGHL00121 is PDE5, with an IC_50_ value of 1.7 nM in enzyme assays using PDE-V (guinea pig, lung).

### Evaluation of OGHL00006, OGHL00169, and OGHL00217 derivatives

From the additional hits identified through the parallel screening approach by the Basel team, namely OGHL00006, OGHL00169, and OGHL00217, 32 related analogues were obtained and tested against NTS. OGHL00087 was not considered further at this stage as it was not active against adult *S. mansoni*, and OGHL00022 was evaluated as described above by the Giessen team.

**Fig. 11:**
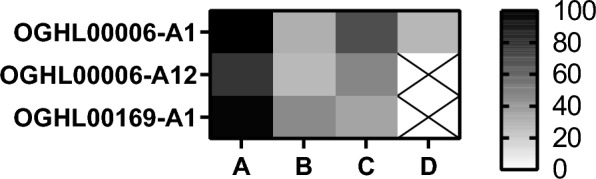
In vitro screening results of 32 analogues of OGHL0006, OGHL00169, and OGHL00217, with effects. NTS and adult *S. mansoni* were treated with concentrations between 0.1 µM and 10 µM by the Basel team. For the heat map, the scale is given for the compound effects from 0% (white) to 100% (black).** A**,** B** Effects on NTS after 72 h of treatment with 10 µM and 1 µM of each compound, respectively.** C**,** D** Effects on couples after 72 h of treatment with 10 µM and 1 µM of each compound, respectively

As shown in Fig. 11, of the 32 derivatives tested, three compounds (OGHL00006-A1, OGHL00006-A12, OGHL00169-A1) showed high activity against NTS (79.1-100%). Of these, only OGHL00006-A1 showed moderate activity of 69% at 10 µM against adult *S. mansoni*, while the other two compounds were not active against this stage.

## Discussion

To meet the challenge of finding alternative drugs to fight schistosomiasis and other parasitic diseases, screening of compound libraries against in vitro-cultured worms is one option because it can support the identification of substances with immediate anti-parasitic effects [36–38]. Hits can serve as starting points for further development. Against this background, the screening of the OGHL against schistosomes is particularly interesting, as this library contains compounds that have reached an early phase of development. The screening approach used by the Giessen team focused on rapidly acting compounds. Specifically, compounds were further investigated that exhibited effects within 24 h after single administration. The goal was to identify potential new drugs or lead structures, which could be further developed as PZQ alternatives. The approach of finding fast-acting compounds aimed at identifying candidates for one-time-application strategies. This is especially important considering the unique context of endemic regions that often face challenges with respect to regular healthcare access and controlled drug administration. In parallel, the screening approach used by the Basel team followed the widely established pre-screening with NTS, and hits were subsequently evaluated on adult worms. Use of established in vitro assays of two independent labs identified various effects on adults and juvenile *S. mansoni* of the Liberian strain [25], which was maintained in both labs.

One incidental observation by the Giessen team upon treating paired adults was that > 30% of the main library compounds affected egg production, of which 23 compounds increased and 56 reduced the number of produced eggs in vitro. This suggests that nearly one-third of the compound library may comprise substances that could target molecules involved in molecular processes controlling egg formation. Indeed, the compounds influencing egg production, among other effects, target signalling and cell cycle-regulating genes (Additional File 1). Earlier studies showed that signal transduction molecules cooperatively operate in a balanced way to regulate egg synthesis; among these molecules were Src kinase (promoting) and TGFβ receptor (reducing) proteins. Therefore, among the compounds that caused an increase in egg production might be candidates targeting TGFβ receptor pathway members and associated molecules, whereas among the compounds showing the opposite effects might be candidates targeting Src kinase pathway members [39, 40]. The egg production-reducing compound OGHL00036, for instance, targets angiotensin II receptors, which have been shown to cooperatively interact with TGFβ receptors [41, 42]. The egg production-inducing compound OGHL00225, for instance, targets the prostaglandin E receptor 2, which is associated with Src kinases regulating the p38 pathway [43]. Compounds reducing—and in the best case inhibiting—egg production are particularly noteworthy. Reduced egg production may not only interfere with the schistosome life cycle and reduce environmental contamination, it may also diminish the pathology of schistosomiasis, which is closely associated with the egg stage [44, 45].

With respect to more immediate effects on worm survival, 61 compounds tested by the Giessen team caused a reduction in worm motility. Among these were two hits from the initial screening approach, OGHL00022 and OGHL00121, both of which exhibited strong antischistosomal effects even at low µM concentrations. Specifically, the EC_50_ values for the motility of paired adults were 15.72 µM after 4 h and 18.77 µM after 24 h for OGHL00022. For OGHL00121, the EC_50_ value after 4 h was 12.75 µM, which was decreased further to 6.297 µM after 24 h. In comparison, EC_50_ values in other studies for PZQ were 0.1 µg/mL at 4 h, 0.05 µg/mL at 72 h [33] and 0.1 µM at 72 h [32]. In contrast to PZQ, which resulted in a motility score of about 0 in the initial approach, the same score was not reached by either compound within 24 h.

In addition to motility, OGHL00022 and OGHL00121 affected stem cell proliferation, as shown by EdU staining. To investigate further potential reasons for reduced egg production after treatment with these substances, CLSM analyses was performed with carmine red-stained worms. This method has been shown to be suitable for the visualization of the integrity of female schistosome gonads, ovary and vitellarium, including the presence, as well as the amount of, stem cell-like oogonia and mature oocytes in female *S. mansoni* [23, 28]. However, except for reduced stem cell numbers, there was no further indication of morphological changes in the female gonads that could explain the observed reduction in egg production. One explanation, however, could be the reduced motility of treated couples, which may have physically influenced egg production-associated molecular processes. In a cell biology context, schistosomiasis has been discussed as a disease caused by/originating from parasite stem cells [34]. Therefore, the effects on stem cells found after treatment with OGHL00022 and OGHL00121 can be considered beneficial properties of both compounds.

The Giessen team also noted that simultaneous treatment with both OGHL00022 and OGHL00121 resulted in additive effects on motility. As evident from the EC_50_ values, OGHL00022 acted quickly, although this effect appeared to be reversible, while OGHL00121 acted more slowly but strongly after 24 h. Considering these features, the double treatment approach appears to be an interesting one because it combines a fast-acting compound with a with a long-acting (‘depot-like’) compound.

The screening by the Giessen team with a focus on fast-acting compounds yielded two hit candidates, OGHL00022 and OGHL00121. Screening by the Basel team also identified OGHL00022 as a hit. In addition, the Basel team provided evidence for the activity of compounds OGHL00006, OGHL00169, and OGHL00217. OGHL00121, identified as a hit by the Giessen team, was not active on NTS, and thus has not been tested against adults by the Basel team. It is worth highlighting that none of the lead compounds in Basel were observed to be fast acting, with only moderate activity (around 50%) observed after 24 h of incubation.

Thus, evaluating the same library using worms of the same *S. mansoni* strain (Liberian), yet using two varying screening cascades in two different labs, led to varying results. Findings in different labs may not always be comparable, which shows the need for some caution when interpreting our findings. One technical reason for the observed discrepancies between the results of the labs could be the different sources and concentrations of FCS/newborn calf serum used as in vitro culture additives, which may have influenced the activities of the compounds. Serum components such as serum albumin and/or the alpha-1 acidic glycoprotein may negatively interact with synthetic compounds such as small molecule inhibitors [46–48]. Our results suggest that compound library screenings might be done in different labs to support the selection of the most reliable candidates for further development.

Despite the above, the Basel and Giessen labs did discover one common hit, OGHL00022, a compound with potentially broad spectrum activity. However, further assays using juvenile worms are required to investigate this. The observed effects of the parental compound were not improved upon by the tested analogues. However, with respect to structure-based activity, there may be additional chemical space for modifying the parental compound in different ways not yet achieved with the analogues used here.

One known human target of OGHL00022 is CX3CR1, a CX3C chemokine receptor, which is also known as fractalkine receptor or G-protein coupled receptor 13. In human ECV-304 cells, CX3CR1 supports leukocyte migration and fulfils both adhesive and chemotactic functions in cell migration [49]. The *S. mansoni* genome is rich in G-protein coupled receptors [50, 51]; however, a first approach of the Giessen team to identify a CX3CR1 orthologue showed no convincing results. So the mode of action within *S. mansoni* remains unclear.

**Table 1:** Overview of the in vitro screening results for OGHL00022 and OGHL00121 of the Giessen team

Compound	OGHL00022	OGHL00121
Structure		
Molecular mass (g/mol)	307.14	502.96
Topological polar surface area (TPSA) (*A*^2^)	60.79	125.47
cLog*P*	3.22	6.23
Pairing stability after 4 h	13%	100%
Pairing stability after 24 h	13%	20%
Attachment after 4 h	37%	100%
Attachment after 24 h	77%	63%
Egg production score	0%	0%
Motility score after 4 h	1.4	3.0
Motility score after 24 h	1.9	1.9

**Table 2:** Structures of the additional hits OGHL00006, OGHL00169, and OGHL00217 found by the Basel team

Compound	OGHL00006	OGHL00169	OGHL00217
Structure			
Molecular mass (g/mol)	394.32	391.38	452.52
TPSA (*A*^2^)	62.68	77.21	79.02
cLog*P*	4.11	3.96	4.45

## Conclusions

In summary, against the background of the urgent need to find alternatives to PZQ, based on the results presented in this study, it appears worthwhile to further explore OGHL00022 by structure activity-based analyses to generate optimized compounds for extended evaluation, including animal experiments.

## Supplementary Information

Additional file1.

## Data Availability

No datasets were generated or analysed during the current study.

## References

[1] World Health Organization. Schistosomiasis. 2023. https://www.who.int/news-room/fact-sheets/detail/schistosomiasis. Accessed 24 Oct 2023.

[2] McManus DP, Dunne DW, Sacko M, Utzinger J, Vennervald BJ, Zhou XN. Schistosomiasis. Nat Rev Dis Prim. 2018;4:13. 10.1038/s41572-018-0013-8. 30093684 10.1038/s41572-018-0013-8

[3] McCreesh N, Nikulin G, Booth M. Predicting the effects of climate change on *Schistosoma mansoni* transmission in eastern Africa. Parasit Vectors. 2015;8:4. 10.1186/s13071-014-0617-0. 25558917 10.1186/s13071-014-0617-0 PMC4297451

[4] Arsuaga M, Díaz-Menéndez M, Gobbi FG. Autochthonous schistosomiasis in Europe: a silent threat. Travel Med Infect Dis. 2022;45:102244. 10.1016/j.tmaid.2021.102244. 34942375 10.1016/j.tmaid.2021.102244

[5] Standley CJ, Mugisha L, Dobson AP, Stothard JR. Zoonotic schistosomiasis in non-human primates: past, present and future activities at the human-wildlife interface in Africa. J Helminthol. 2012;86:131–40. 10.1017/S0022149X12000028. 22269859 10.1017/S0022149X12000028

[6] Rollinson D, editor. The biology of schistosomes: from genes to latrines. London: Academic Pr.; 1987.

[7] Grevelding CG. Schistosoma. Curr Biol. 2004;14:R545. 10.1016/j.cub.2004.07.006. 15268869 10.1016/j.cub.2004.07.006

[8] Colley DG, Bustinduy AL, Secor WE, King CH. Human schistosomiasis. Lancet. 2014;383:2253–64. 10.1016/S0140-6736(13)61949-2. 24698483 10.1016/S0140-6736(13)61949-2 PMC4672382

[9] Gryseels B, Polman K, Clerinx J, Kestens L. Human schistosomiasis. Lancet. 2006;368:1106–18. 10.1016/S0140-6736(06)69440-3. 16997665 10.1016/S0140-6736(06)69440-3

[10] Grimes JET, Croll D, Harrison WE, Utzinger J, Freeman MC, Templeton MR. The roles of water, sanitation and hygiene in reducing schistosomiasis: a review. Parasit Vectors. 2015;8:156. 10.1186/s13071-015-0766-9. 25884172 10.1186/s13071-015-0766-9 PMC4377019

[11] Merck KGaA. Merck stellt 1,5-milliardste Tablette Praziquantel zur Behandlung von Bilharziose bereit. 2022. https://www.merckgroup.com/de/news/praziquantel-tablet-donation-24-01-2022.html. Accessed 26 Oct 2023.

[12] Kokaliaris C, Garba A, Matuska M, Bronzan RN, Colley DG, Dorkenoo AM, et al. Effect of preventive chemotherapy with praziquantel on schistosomiasis among school-aged children in sub-Saharan Africa: a spatiotemporal modelling study. Lancet Infect Dis. 2022;22:136–49. 10.1016/S1473-3099(21)00090-6. 34863336 10.1016/S1473-3099(21)00090-6 PMC8695385

[13] Doenhoff MJ, Kusel JR, Coles GC, Cioli D. Resistance of *Schistosoma mansoni* to praziquantel: is there a problem? Trans R Soc Trop Med Hyg. 2002;96:465–9. 10.1016/s0035-9203(02)90405-0. 12474468 10.1016/s0035-9203(02)90405-0

[14] Melman SD, Steinauer ML, Cunningham C, Kubatko LS, Mwangi IN, Wynn NB, et al. Reduced susceptibility to praziquantel among naturally occurring Kenyan isolates of *Schistosoma mansoni*. PLoS Negl Trop Dis. 2009;3:e504. 10.1371/journal.pntd.0000504. 19688043 10.1371/journal.pntd.0000504 PMC2721635

[15] Wang W, Wang L, Liang YS. Susceptibility or resistance of praziquantel in human schistosomiasis: a review. Parasitol Res. 2012;111:1871–7. 10.1007/s00436-012-3151-z. 23052781 10.1007/s00436-012-3151-z

[16] Cioli D, Pica-Mattoccia L, Basso A, Guidi A. Schistosomiasis control: praziquantel forever? Mol Biochem Parasitol. 2014;195:23–9. 10.1016/j.molbiopara.2014.06.002. 24955523 10.1016/j.molbiopara.2014.06.002

[17] Bergquist R, Utzinger J, Keiser J. Controlling schistosomiasis with praziquantel: how much longer without a viable alternative? Infect Dis Poverty. 2017;6:74. 10.1186/s40249-017-0286-2. 28351414 10.1186/s40249-017-0286-2 PMC5371198

[18] Lin CS, Chow S, Lau A, Tu R, Lue TF. Identification and regulation of human PDE5A gene promoter. Biochem Biophys Res Commun. 2001;280:684–92. 10.1006/bbrc.2000.4220. 11162575 10.1006/bbrc.2000.4220

[19] O’Connor T, Borsig L, Heikenwalder M. CCL2-CCR2 signaling in disease pathogenesis. Endocr Metab Immun Disord Drug Target. 2015;15:105–18. 10.2174/1871530315666150316120920. 10.2174/1871530315666150316120920 25772168

[20] Alvarez BV, Villa-Abrille MC. Mitochondrial NHE1: a newly identified target to prevent heart disease. Front Physiol. 2013;4:152. 10.3389/fphys.2013.00152. 23825461 10.3389/fphys.2013.00152 PMC3695379

[21] Liu YN, Zhang H, Zhang L, Cai TT, Huang DJ, He J, et al. Sphingosine 1 phosphate receptor-1 (S1P1) promotes tumor-associated regulatory T cell expansion: leading to poor survival in bladder cancer. Cell Death Dis. 2019;10:50. 10.1038/s41419-018-1298-y. 30718502 10.1038/s41419-018-1298-y PMC6362099

[22] Andrews P, Thomas H, Pohlke R, Seubert J. Praziquantel. Med Res Rev. 1983;3:147–200. 10.1002/med.2610030204. 6408323 10.1002/med.2610030204

[23] Beckmann S, Quack T, Burmeister C, Buro C, Long T, Dissous C, et al. *Schistosoma mansoni*: signal transduction processes during the development of the reproductive organs. Parasitology. 2010;137:497–520. 10.1017/S0031182010000053. 20163751 10.1017/S0031182010000053

[24] Mughal MN, Grevelding CG, Haeberlein S. First insights into the autophagy machinery of adult *Schistosoma mansoni*. Int J Parasitol. 2021;51:571–85. 10.1016/j.ijpara.2020.11.011. 33713647 10.1016/j.ijpara.2020.11.011

[25] Grevelding CG. The female-specific W1 sequence of the Puerto Rican strain of *Schistosoma mansoni* occurs in both genders of a Liberian strain. Mol Biochem Parasitol. 1995;71:269–72. 10.1016/0166-6851(94)00058-u. 7477111 10.1016/0166-6851(94)00058-u

[26] Hahnel S, Lu Z, Wilson RA, Grevelding CG, Quack T. Whole-organ isolation approach as a basis for tissue-specific analyses in *Schistosoma mansoni*. PLoS Negl Trop Dis. 2013;7:e2336. 10.1371/journal.pntd.0002336. 23936567 10.1371/journal.pntd.0002336 PMC3723596

[27] Lombardo FC, Pasche V, Panic G, Endriss Y, Keiser J. Life cycle maintenance and drug-sensitivity assays for early drug discovery in *Schistosoma mansoni*. Nat Protoc. 2019;14:461–81. 10.1038/s41596-018-0101-y. 30610241 10.1038/s41596-018-0101-y

[28] Beckmann S, Buro C, Dissous C, Hirzmann J, Grevelding CG. The Syk kinase SmTK4 of *Schistosoma mansoni* is involved in the regulation of spermatogenesis and oogenesis. PLoS Pathog. 2010;6:e1000769. 10.1371/journal.ppat.1000769. 20169182 10.1371/journal.ppat.1000769 PMC2820527

[29] Beutler M, Harnischfeger J, Weber MHW, Hahnel SR, Quack T, Blohm A, et al. Identification and characterisation of the tegument-expressed aldehyde dehydrogenase SmALDH_312 of *Schistosoma mansoni*, a target of disulfiram. Eur J Med Chem. 2023;251:115179. 10.1016/j.ejmech.2023.115179. 36948075 10.1016/j.ejmech.2023.115179

[30] Hahnel S, Quack T, Parker-Manuel SJ, Lu Z, Vanderstraete M, Morel M, et al. Gonad RNA-specific qRT-PCR analyses identify genes with potential functions in schistosome reproduction such as SmFz1 and SmFGFRs. Front Genet. 2014;5:170. 10.3389/fgene.2014.00170. 24959172 10.3389/fgene.2014.00170 PMC4050651

[31] Preibisch S, Saalfeld S, Tomancak P. Globally optimal stitching of tiled 3D microscopic image acquisitions. Bioinformatics. 2009;25:1463–5. 10.1093/bioinformatics/btp184. 19346324 10.1093/bioinformatics/btp184 PMC2682522

[32] Patra M, Ingram K, Leonidova A, Pierroz V, Ferrari S, Robertson MN, et al. In vitro metabolic profile and *in vivo* antischistosomal activity studies of (η(6)-praziquantel)Cr(CO)3 derivatives. J Med Chem. 2013;56:9192–8. 10.1021/jm401287m. 24219617 10.1021/jm401287m

[33] Meister I, Ingram-Sieber K, Cowan N, Todd M, Robertson MN, Meli C, et al. Activity of praziquantel enantiomers and main metabolites against *Schistosoma mansoni*. Antimicrob Agent Chemother. 2014;58:5466–72. 10.1128/AAC.02741-14. 10.1128/AAC.02741-14 PMC4135865 24982093

[34] Wendt GR, Collins JJ. Schistosomiasis as a disease of stem cells. Curr Opin Genet Dev. 2016;40:95–102. 10.1016/j.gde.2016.06.010. 27392295 10.1016/j.gde.2016.06.010 PMC5135665

[35] Collins JJ, Wendt GR, Iyer H, Newmark PA. Stem cell progeny contribute to the schistosome host-parasite interface. Elife. 2016;5:e12473. 10.7554/eLife.12473. 27003592 10.7554/eLife.12473 PMC4841766

[36] Caldwell N, Afshar R, Baragaña B, Bustinduy AL, Caffrey CR, Collins JJ, et al. Perspective on schistosomiasis drug discovery: highlights from a schistosomiasis drug discovery workshop at Wellcome Collection, London, September 2022. ACS Infect Dis. 2023;9:1046–55. 10.1021/acsinfecdis.3c00081. 37083395 10.1021/acsinfecdis.3c00081 PMC10186373

[37] Ferreira LLG, de Moraes J, Andricopulo AD. Approaches to advance drug discovery for neglected tropical diseases. Drug Discov Today. 2022;27:2278–87. 10.1016/j.drudis.2022.04.004. 35398562 10.1016/j.drudis.2022.04.004

[38] Zajíčková M, Nguyen LT, Skálová L, Raisová Stuchlíková L, Matoušková P. Anthelmintics in the future: current trends in the discovery and development of new drugs against gastrointestinal nematodes. Drug Discov Today. 2020;25:430–7. 10.1016/j.drudis.2019.12.007. 31883953 10.1016/j.drudis.2019.12.007

[39] Buro C, Oliveira KC, Lu Z, Leutner S, Beckmann S, Dissous C, et al. Transcriptome analyses of inhibitor-treated schistosome females provide evidence for cooperating Src-kinase and TGFβ receptor pathways controlling mitosis and eggshell formation. PLoS Pathog. 2013;9:e1003448. 10.1371/journal.ppat.1003448. 23785292 10.1371/journal.ppat.1003448 PMC3681755

[40] Knobloch J, Kunz W, Grevelding CG. Herbimycin A suppresses mitotic activity and egg production of female *Schistosoma mansoni*. Int J Parasitol. 2006;36:1261–72. 10.1016/j.ijpara.2006.06.004. 16844129 10.1016/j.ijpara.2006.06.004

[41] Ehanire T, Ren L, Bond J, Medina M, Li G, Bashirov L, et al. Angiotensin II stimulates canonical TGFβ signaling pathway through angiotensin type 1 receptor to induce granulation tissue contraction. J Mol Med (Berl). 2015;93:289–302. 10.1007/s00109-014-1211-9. 25345602 10.1007/s00109-014-1211-9 PMC4334749

[42] Nataatmadja M, West J, Prabowo S, West M. Angiotensin II receptor antagonism reduces transforming growth factor beta and smad signaling in thoracic aortic aneurysm. Ochsner J. 2013;13:42–8. 23532685 PMC3603187

[43] Chun KS, Shim M. EP2 induces p38 phosphorylation via the activation of Src in HEK 293 cells. Biomol Ther (Seoul). 2015;23:539–48. 10.4062/biomolther.2015.043. 26535079 10.4062/biomolther.2015.043 PMC4624070

[44] Hams E, Aviello G, Fallon PG. The schistosoma granuloma: friend or foe? Front Immunol. 2013;4:89. 10.3389/fimmu.2013.00089. 23596444 10.3389/fimmu.2013.00089 PMC3625856

[45] Pagán AJ, Ramakrishnan L. The formation and function of granulomas. Annu Rev Immunol. 2018;36:639–65. 10.1146/annurev-immunol-032712-100022. 29400999 10.1146/annurev-immunol-032712-100022

[46] Huang Z, Ung T. Effect of alpha-1-acid glycoprotein binding on pharmacokinetics and pharmacodynamics. Curr Drug Metab. 2013;14:226–38. 23092311

[47] Filip Z, Jan K, Vendula S, Jana KZ, Kamil M, Kamil K. Albumin and α1-acid glycoprotein: old acquaintances. Expert Opin Drug Metab Toxicol. 2013;9:943–54. 10.1517/17425255.2013.790364. 23621565 10.1517/17425255.2013.790364

[48] Beckmann S, Long T, Scheld C, Geyer R, Caffrey CR, Grevelding CG. Serum albumin and α-1 acid glycoprotein impede the killing of *Schistosoma mansoni* by the tyrosine kinase inhibitor Imatinib. Int J Parasitol Drugs Drug Resist. 2014;4:287–95. 10.1016/j.ijpddr.2014.07.005. 25516839 10.1016/j.ijpddr.2014.07.005 PMC4266805

[49] Imai T, Hieshima K, Haskell C, Baba M, Nagira M, Nishimura M, et al. Identification and molecular characterization of fractalkine receptor CX3CR1, which mediates both leukocyte migration and adhesion. Cell. 1997;91:521–30. 10.1016/s0092-8674(00)80438-9. 9390561 10.1016/s0092-8674(00)80438-9

[50] Hahnel S, Wheeler N, Lu Z, Wangwiwatsin A, McVeigh P, Maule A, et al. Tissue-specific transcriptome analyses provide new insights into GPCR signalling in adult *Schistosoma mansoni*. PLoS Pathog. 2018;14:e1006718. 10.1371/journal.ppat.1006718. 29346437 10.1371/journal.ppat.1006718 PMC5773224

[51] Kamara IK, Thao JT, Kaur K, Wheeler NJ, Chan JD. Annotation of G-protein coupled receptors in the genomes of parasitic blood flukes. MicroPubl Biol. 2023. 10.17912/micropub.biology.000704. eCollection 2023. 36713056 10.17912/micropub.biology.000704 PMC9874797

