# Supplementary material for: Merck Open Global Health Library in vitro screening against Schistosoma mansoni identified two new substances with antischistosomal activities for further development
Source: Parasit Vectors. 2025 Feb 4;18:40. doi: 10.1186/s13071-024-06648-0 (PMC11796224; doi:10.1186/s13071-024-06648-0)
Supplement: Supplementary file 1 — Additional file1. [file 13071_2024_6648_MOESM1_ESM.pdf]

Reduction of egg production	Target	Increase of egg production	Target
OGHL00225	EP2	OGHL00116	PDE5
OGHL00226	EP2	OGHL00187	CCR2
OGHL00131	TIE2	OGHL00189	CCR2
OGHL00243	EP2	OGHL00014	PDE3
OGHL00084	PDE5	OGHL00047	NHE1
OGHL00164	S1P1	OGHL00095	PDE5
OGHL00212	GSM1	OGHL00207	OGA
OGHL00244	Sm.TRPMpzq	OGHL00080	PDE5
OGHL00133	LRRK2	OGHL00128	GLEPP-1
OGHL00245	Sm.TRPMpzq	OGHL00160	S1P1
OGHL00006	CX3CR1	OGHL00114	PDE5
OGHL00022	CX3CR1	OGHL00059	NHE1
OGHL00134	LRRK2	OGHL00139	S1P1
OGHL00182	M1	OGHL00161	S1P1
OGHL00198	CCR2	OGHL00066	PDE4
OGHL00214	GSM1	OGHL00104	PDE4
OGHL00230	M1	OGHL00090	PDE5
OGHL00246	EP2	OGHL00154	S1P1
OGHL00039	NHE1	OGHL00155	S1P1
OGHL00103	PDE5	OGHL00012	PDE3
OGHL00231	EP2	OGHL00142	S1P1
OGHL00216	GSM1	OGHL00143	S1P1
OGHL00025	Ca(2+)-sensitizer	OGHL00016	CDC25A
OGHL00089	PD5		
OGHL00121	PD5		
OGHL00169	TBK1		
OGHL00138	LRRK2		
OGHL00250	TBK1		
OGHL00108	PDE5		
OGHL00124	CXCR5		
OGHL00236	EP2		
OGHL00062	NHE1		
OGHL00078	PDE5		

OGHL00027	K-ATP Potassium Channel		
OGHL00219	GSM1		
OGHL00093	PDE5		
OGHL00237	EP2		
OGHL00126	GLEPP-1		
OGHL00224	MGLUR3		
OGHL00241	EP2		
OGHL00034	AT1R		
OGHL00050	NHE1		
OGHL00036	AT1R		
OGHL00021	CDC25B		
OGHL00053	NHE1		
OGHL00101	PDE5		
OGHL00248	EP2		
OGHL00043	PDE5		
OGHL00092	PDE5		
OGHL00061	NHE1		
OGHL00109	PDE5		
OGHL00094	NHE1		
OGHL00110	CX3CR1		
OGHL00079	PDE5		
OGHL00208	GSM1		
OGHL00240	EP2		

Supplementary Table 1. Overview of all library compounds (OGHL_X Column) that affected egg production. Potential targets of the appropriate compounds are given in abbreviated form (Target column). AT1R - Angiotensin II receptor type 1; Ca(2+)-sensitizer - Calcium sensitizer; CCR2 - C-C chemokine receptor type 2; CDC25A - M-phase inducer phosphatase 1; CDC25B - M-phase inducer phosphatase 2; CX3CR1 - C-X3-C motif chemokine receptor 1; CXCR5 - CXC motif chemokine receptor 5; EP2 - Prostglandin E receptor 2 (subtype); GLEPP-1 - Membrane protein-tyrosine phosphatase; GSM1 - Geniospasm 1; K-ATP Potassium Channel - ATP-sensitive potassium channel; LRRK2 - Leucine-rich repeat kinase 2; M1 - Matrix protein of the influenza virus; MGLUR3 - Metabotropic glutamate receptor 3; NHE1 - Sodium-hydrogen antiporter 1; OGA - O-GlcNAcase; PD5 - Programmed cell death 5; PDE3 - Phosphodiesterase 3; PDE4 - Phosphodiesterase 4; PDE5 - Phosphodiesterase 5; S1P1 - Sphingosine-1-phosphate receptor 1; Sm.TRPMpzq - Transient receptor channel in *S. mansoni*; TBK1 - TANK-binding kinase 1; TIE2 - Angiopoietin-1 Receptor
